# Epidemiology of Ixodid tick infestation and tick‐borne haemopathogens in small ruminant from Enarje Enawuga, North Western Ethiopia

**DOI:** 10.1002/vms3.1080

**Published:** 2023-01-30

**Authors:** Habtamu Tamrat, Wudu Tagel, Negus Belayneh

**Affiliations:** ^1^ School of Animal Science and Veterinary Medicine, College of Agriculture and Environmental Sciences Bahir Dar University Bahir Dar Ethiopia; ^2^ Livestock Resource Development Office Debre Werk Ethiopia; ^3^ Andasa Livestock Research Center Bahir Dar Ethiopia

**Keywords:** Ethiopia, Ixodid tick, prevalence, risk factors, small ruminant, tick‐borne haemopathogen

## Abstract

**Background:**

Small ruminant playing significant economic development and cultural roles for smallholders and reared in different agroecological systems in Ethiopia. However, ticks and tick‐borne haemopathogens are major threats to the health of small ruminants leading to loss of production and productivity in Ethiopia especially in north‐western Ethiopia, due to climate and agroecological system favourable to the tick survival in the area.

**Objectives:**

The objectives of this study were to determine the prevalence of Ixodid tick infestation and tick‐borne haemopathogens, to identify the existing Ixodid tick species and to investigate major risk factors associated with tick infestation and tick‐borne haemopathogens.

**Methods:**

A cross sectional study was conducted on 396 small ruminants (sheep =198 and goats = 198). Ticks were identified to the species level according to their morphological key structures. Thin blood smear were prepared and laboratoricaly examined to investigate tick‐borne haemopathogens. Logistic regression was employed to assess the association between potential risk factors with tick infestation and tick‐borne haemopathogens.

**Results:**

The overall prevalence of tick infestation and tick‐borne haemopathogens were 47.22% and 4.29%, respectively. Age, species, body condition, agroecological system and season were found significantly (*p* < 0.05) associated with tick infestation. Season was found to be significantly (*p* < 0.05) associated with occurrence of tick‐borne haemopathogen. *Amblyomma variegatum* (18.2%) and *Rhipicephalus evertsi evertsi* (13.4%) were the predominant tick species encountered. *Anaplasma ovis*, *Anaplasma marginale* and *Babesia ov*is were prevalent tick‐borne haemopathogens.

**Conclusions:**

The present study reported that there was higher tick infestation and tick‐borne haemopathogens infections on small ruminants in the study area. Therefore, based on tick epidemiology, a strategic tick control programs were needed in this region. Further detailed studies on the role of Ixodid tick species in causing tick‐borne diseases in small ruminants and their economic impact on livelihoods should be conducted.

## Graphical abstract

A cross sectional study was conducted on 396 small ruminants (sheep = 198 and goats = 198). Ticks were identified to the species level according to their morphological key structures under a stereomicroscope. Thin blood smear were prepared and laboratoricaly examined to investigate tick‐borne haemopathogens. The overall prevalence of tick infestation and tick‐borne haemopathogens in small ruminants in this study were 47.22% and 4.29%, respectively. *Amblyomma variegatum* (18.2%) and *Rhipicephalus evertsi evertsi* (13.4%) were the predominant tick species encountered. *Anaplasma ovis, Anaplasma marginale* and *Babesia ov*is were prevalent tick‐borne haemopathogens.

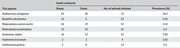

## 1 INTRODUCTION

Small ruminants are an important aspect of livestock in Sub‐Saharan Africa where they are primarily kept for immediate income, milk, meat, wool, manure and saving as a living bank asset. Small ruminants also serve a variety of social and cultural functions that varies among different cultures, socioeconomies, agroecologies and locations in tropical and sub‐tropical Africa (World Bank, 2006). The livestock share of agricultural output in Ethiopia is about 40%, which making it an important sector for the country's economy and contributing significantly to gross domestic product (Tesfaheywet & Tadesse, 2012). Moreover, small ruminants provide the country with export goods such as live animals and skins to help the government earn hard currency; in fact, sheep and goat skins are among the most popular export commodities (Kleemann, 2008; Yami & Merke, 2009; USAID, 2013).

In Ethiopia, skin diseases of small ruminants are responsible for significant economic losses particularly to the skin and hide export sectors due to various defects (Girma & Yami, 2008). Ticks, lice, fleas and mites are some of the most common external parasites that create visible skin lesions (Mullen & Durden, 2002; Pugh, 2002). Ticks can cause mechanical damage, anaemia, toxicity and paralysis in severe infestation and act as vector for tick‐borne infections (Walker et al., 2003; Wall & Shearer, 2001). There are more than 850 different species of ticks in the globe, with 60 of them found in Africa. In Ethiopia there are 47 tick species, and the majority of them are important vectors and disease‐causing agents, in addition to having a negative impact on skin and hide production. Ticks and tick‐borne infections are the third most common parasitic diseases in Ethiopia after trypanosomes and endoparasitism, causing significant losses to the livestock sector (Kassa, 2005).

In Ethiopia, hides are used 45% of the time, goat skin is used 75% of the time, and sheep skin is used 97% of the time, with predicted off take of 33%, 35% and 7% for sheep, goats and cattle, respectively (Kassa, 2006). However, in recent years, this rank has been downgraded to the fifth level, owing to the rejection and downgrading of hides, as well as skin abnormalities caused by external parasites (Kassa, 2006). Ticks are responsible for severe losses directly by blood sucking and indirectly by disease transmission (Morel, 1980). Tick bite may be directly debilitating to domestic animals, causing mechanical damage, irritation, inflammation and hypersensitivity and, when present in large numbers feeding may cause anaemia and loss of production. Some species cause tick paralysis and the others will elaborate toxins other than those causing paralysis. A heavy tick load causes enough concern to interfere with feeding, which may lead to loss of production and weight loss (Radostits et al., 2000). Although many similar studies have been conducted to determine the prevalence, species composition and associated risk factors related to tick infestation and tick‐borne haemopathogens infections on small ruminants from different areas of Ethiopia, it should be noted that Ethiopia is a large country with a huge number of livestock populations and, therefore, most studies only target specific areas and not the whole country. Furthermore, there is no known research conducted in the past and no published information was reported regarding Ixodid ticks' infestation and tick‐borne haemopathogens' infection in small ruminants from the study region. Therefore, the objectives of this study were to determine the prevalence of tick infestations and tick‐borne haemopathogens' infections on small ruminants, to identify the existing Ixodid tick species in our investigated area and to analyse the potential risk factors associated with tick infestations and the circulation of tick‐borne haemopathogens in the study area.

## 2 MATERIALS AND METHODS

### 2.1 Study area

The study was conducted in Enarji Enawuga District, Amhara Regional State, Ethiopia. Enarji Enawuga is located 291 km from Addis Ababa, the capital city of Ethiopia. It is located at 10°50'N latitude and 38° 05'E longitudes, with an average temperature range of 22.5–25°C and a mean annual rainfall range of 700–2000 mm. The district covers an estimated area of 96,095 hectares. According to (EEWARDO, 2013), the district is divided into three agroecological systems, with 30% highland, 50% midland and 20% lowland. Topographically, it has a lowest point of 1100 m above sea level and a peak elevation of 3200 masl. In this district there are 80,820 cattle, 46,528 sheep, 44,388 goats, 24,976 horses and 32,679 poultry (EEWARDO, 2013).

### 2.2 Study population

The study populations were small ruminants (sheep and goats) kept under individual households with varying ages, sexes and body conditions.

### 2.3 Study design and sampling procedure

A cross sectional study design was employed. Information about agroecological systems and season were recorded. The study animals were categorised into three age groups as young, adult and old (ESGPIP, 2010). The body condition score was employed after categorising the animals into poor, medium and good. Accordingly, sex, age, and agroecological system were considered as other risk factors (CSA, 2013). Six kebeles from the district were selected by purposive sampling technique based on the representativeness of the three agroecological systems. The study animals which were not treated with any treatments one month prior to sample collection were selected using simple random sampling method.

### 2.4 Sample size determination

The sample size required for this study was determined according to Thrusfield (2007). Sample size was estimated assuming a 50% of expected prevalence, at 95% confidence interval and 5% of required absolute precision as there is no documented information about Ixodid tick infestation and tick‐borne haemopathogens on small ruminants in study area. Thus, a total of 396 small ruminants (198 sheep and 198 goats) were sampled for this study.

### 2.5 Data collection

#### 2.5.1 Tick sample collection

Small ruminants were restrained properly prior to tick sample collection. Ticks, which were visible and found attached on the host, were collected through careful inspection and application of forceps from the skin of each study animal from different predilection sites (eye, ear, teat, scrotum, udder, neck, sternum, tail and anus). Collected ticks were preserved in universal bottles containing 70% ethyl alcohol for further identification. The samples were labelled and then transported to Bahir Dar animal health investigation and diagnostic laboratory, parasitological laboratory.

#### 2.5.2 Blood smear preparation and staining procedure

Blood sample collection was done after proper restraining of the animal according to Urquhart et al. (1996). After marginal ear vein was punctured and blood oozed, it was taken directly by microscopic slide and thin blood smear was made on the other clean microscopic slide and fixed by methanol after dried in the air for 3–5 min and transported to the Bahir Dar Animal Health Diagnostic and Investigation Laboratory (BAHDIL), parasitological laboratory as soon as possible. All samples were clearly labelled with the species, sex, age and body condition score of sheep and goats. After transported to BAHDIL parasitological laboratory, the fixed blood smears were stained with working solution of Giemsa for 30–45 min and washed with tap water to remove extra stain and was air dried (Benjamin, 2005; Cheesbrough, 1999).

#### 2.5.3 Tick identification

Species level tick identification were done according to their morphological key structures such as shape of scutum, leg colour, scutum ornamentation, body grooves, punctuations, basis capitulum, coxaes and ventral plates. During tick identification in the laboratory the sample were put on Petri dish and adult ticks were identified to species level under a stereomicroscope using the standard identification keys (Houseman, 2013; Walker et al., 2014).

#### 2.5.4 Microscopic examination of haemopathogens

Fixed smears were examined by using compound microscope under oil immersion lens. During laboratorial examination identification of all tick‐borne haemopathogens of small ruminants were considered. *Anaplasma* species were identified on the basis of its morphology Bowmann (2009) and the number of infected erythrocytes. *Babesia* species have been identified morphologically based on the size of their merozoites when viewed by light microscopy in stained blood smears (Laetitia et al., 2017).

#### 2.5.5 Data management and analysis

All field and laboratory examination data were organised and feed into Microsoft Excel spread sheets and coded appropriately and analysed using STATA statistical software version 14.0. The data were summarised using descriptive statistics and displayed by tables and graphs. Calculations of tick infestation prevalence was done by dividing number of small ruminants infested by ticks to total number of surveyed small ruminants (396). Likewise tick‐borne haemopathogen infection prevalence = number of small ruminants with positive blood smears to tick‐borne haemopathogen /total number of surveyed small ruminants. Logistic regression was employed to analyse the degree of association of outcome (presence or absence of tick and tick‐borne haemopathogens) as a function of various potential risk factors. Odds ratio was used to quantify the association among the factors with the presence of tick infestation and presence of tick‐borne haemopathogens. Effects were considered as statistically significant in all cases if *p* < 0.05.

## 3 RESULTS

### 3.1 Prevalence of Ixodid tick infestation and tick‐borne haemopathogens

From a total of 396 small ruminants examined in this study, 109 (55.05%) sheep and 78 (39.39%) goats were found infested with ticks. The overall prevalence of Ixodid tick infestation was 47.22% (Table 1). The prevalence of tick‐borne haemopathogens in sheep and goat was 5.05% and 3.54%, respectively with over all prevalence of 4.29% (Table 2). In the current study, three tick‐borne haemopathogens were identified. *Anaplasma ovis* was the most frequently encounter species. The prevalence of tick‐borne haemopathogens with age, body condition, agroecological systems and season was displayed in Table 3.

**TABLE 1:** Overall prevalence of Ixodid tick infestation on small ruminants

Small ruminant	*N*	No. of animals infested	Prevalence (%)
Sheep	198	109	55.05
Goat	198	78	39.39
Total	396	187	47.22

*N* = number of sampled animals.

**TABLE 2:** Overall prevalence of tick‐borne haemopathogens on small ruminants

Small ruminant	*N*	Number infected animals	Prevalence %
Sheep	198	10	5.05
Goat	198	7	3.54
Total	396	17	4.29

*N* = number of sampled animals.

**TABLE 3:** Prevalence of tick‐borne haemopathogens with different associated risk factors in the district

		Tick‐borne haemopathogens				
Risk factors	Categories (*n*)	*B. ovis*	*A. ovis*	*A. mareginale*	No. of animals infected	Prevalence (%)
Species	Sheep (198)	3	4	3	10	5.05
	Goat (198)	1	6	0	7	3.53
Age	Young (121)	1	1	0	2	1.7
	Adult (167)	2	4	1	7	4.2
	Old (108)	1	5	2	8	7.4
Body condition	Poor (162)	1	6	2	9	5.6
	Medium (121)	1	3	0	1	0.8
	Good (113)	2	1	1	2	1.8
Season	Dry (198)	0	3	1	4	2.02
	Wet (198)	4	7	2	13	6.5
Agroecology	Low land (132)	0	1	0	1	0.8
	Mid land (132)	4	6	1	11	8.3
	High land (132)	0	3	2	5	3.7

*n* = numbers of animals in each category, *B. ovis = Babesia ovis, A. ovis = Anaplasma ovis, A. marginale = Anaplasma marginale*.

### 3.2 Identification of Ixodid tick species

A total of seven tick species were identified. *Amblyomma variegatum* (18.2%) and *Rhipicephalus evertsi* (13.4%) were the predominant tick species encountered in the study area. The distribution of other tick species was indicated (Table 4).

**TABLE 4:** Prevalence of Ixodid tick species isolated in the study area

	Small ruminants			
Tick species	Sheep	Goats	No. of animals infested	Prevalence (%)
*Amblyomma variegatum*	42	30	72	18.2
*Boophilus decoloratus*	16	6	22	5.56
*Rhipicephalus evertsi evertsi*	34	19	53	13.4
*Rhipicephalus praetextatus*	15	10	25	6.3
*Hyalomma rufipes*	16	15	31	7.83
*Hyalomma truncatum*	2	2	4	1.01
*Amblyomma gemma*	5	8	13	3.3

### 3.3 Association of potential risk factors with Ixodid tick infestation

A total of six potential risk factors were tested using multivariate logistic regression. Accordingly species, age, body condition score, agroecological systems and season were statistically significant (*p* < 0.05) risk factors for tick infestation. Keeping the effect of other variables constant, the odds of tick infestation in old aged was 4.71 times more likely than their younger counter parts. Similarly, the odds of tick infestation on small ruminant in wet season were 3.68 times higher than the dry season. Associations of species, sex, body condition and agroecological systems with tick infestation on small ruminant were indicated (Table 5).

**TABLE 5:** Potential risk factors significantly associated to small ruminant Ixodid tick infestation using multivariate logistic regression analysis

Variables	Categories	*N*	No. of animals infested	Prevalence (%)	OR	95% CI	*P* Value
Species	Goat*	198	78	39.39			
	Sheep	198	109	55.05	0.51	0.29–0.89	0.02
Age	Young	121	20	16.53			
	Adult	167	77	46.11			
	Old	108	90	83.33	4.71	3.02–7.3	0.00
Sex	Male*	130	37	28.46			
	Female	266	150	56.39	0.72	0.40–1.31	0.29
BCS	Good*	113	22	19.3			
	Medium	121	43	35.54			
	Poor	162	122	75.78	0.25	0.17–0.37	0.00
Agroecology	Low land	132	47	35.61			
	High land*	132	57	43.18			
	Midland	132	83	62.88	2.34	1.63–3.35	0.00
Season	Dry*	198	66	33.33			
	Wet	198	121	61.11	3.68	2.08–6.52	0.00

* Reference variable.

*N* = number of sampled animals, OR = odd ratio, CI = confidence interval.

### 3.4 Association of potential risk factors with tick‐borne haemopathogens

A total of six potential risk factors were tested using multivariate logistic regression. Season was significantly associated (*p* < 0.05) with tick‐borne haemopathogens. In the current study, the odd of tick‐borne haemopathogens in wet season was 3.59 times higher than the dry season. Similarly, the odd of tick‐borne haemopathogens on small ruminant in old aged was 1.62 times higher than the young aged. The odds of tick‐borne haemopathogens with species, body condition, sex and agroecological systems were displayed in (Table 6).

**TABLE 6:** Potential risk factors significantly associated to small ruminant tick‐borne haemopathogens by multivariate logistic regression analysis

Variable	Categories	*N*	No of infected	Prevalence (%)	OR	95% CI	*p* Value
Species	Goat*	198	10	5.05			
	Sheep	198	7	3.54	0.66	0.23–1.86	0.43
Age	Young*	121	1	0.83			
	Adult	167	8	4.8			
	Old	108	8	7.41	1.62	0.75–3.49	0.22
Sex	Male*	130	3	2.31			
	Female	266	14	5.26	0.67	0.16–2.69	0.57
Bcs	Good*	113	3	2.65			
	Medium	121	5	4.13			
	Poor	162	9	5.6	1.04	0.54–2.03	0.90
Agroecology	Low land*	132	1	0.76			
	Midland	132	11	8.3	1.33	0.72–2.48	0.35
	High land	132	5	3.79			
Season	Dry*	198	4	2.02			
	Wet	198	13	6.57	3.59	1.07–12.08	0.04

* Reference variable.

*N* = number of sampled animals, OR = odd ratio, CI = confidence interval.

## 4 DISCUSSION

The current finding revealed that tick infestation is a widespread and most important constraint of sheep and goats in the study area. This result is in line with report by Abebe et al. (2011) in selected districts of Tigray region, Ethiopia with an overall prevalence of tick infestation of 52.3% in sheep and goats. However, the current study had lower prevalence than reports of Abunna et al. (2009) in sheep 89.9% and in goats 87.5% at Miesso district of Oromia regional State; Abunna et al. (2012) who reported overall prevalence of tick infestation of 76.50% in Bedele district, Oromia Region, Ethiopia; Eyob and Matios (2014) who reported a prevalence of 97.58% in goats and 69.86% in sheep in Borana pastoral area, Southern range lands of Ethiopia; Jafer et al. (2017) in East Harerghe with an overall prevalence of 72.39%. This result is higher than the reports of Sertse and Wosene (2007) in North East Ethiopia with a prevalence of 3.4% and 22.2% in goats and sheep, respectively; Yacob et al. (2008) in and around Wolaita Soddo, Southern part of Ethiopia reported a prevalence of 18.6% in goats and 31.8% in sheep. The difference in the prevalence among reports might be due to the geographical difference, breed difference and season of the study period. There was statistically significant difference (*p* < 0.05) of tick infestation between sheep and goats. This difference might be due to the difference in feeding and self‐grooming behaviours of sheep.

There was statistically significant (*p* < 0.05) difference of prevalence of tick infestation and age of small ruminants, young (16.5%), adult (46.1%) and old (83.3%). This result is in agreement with reports of Yacob et al. (2008) who reported 15% and 53% prevalence of ectoparasites in young and adult sheep and goats, respectively; Kedir and Petros (2015) with the infestation rate of 36.8% and 83.3% in young and old age. However, the current result disagrees with Yonas et al. (2020) in Somali Region, Ethiopia who reported prevalence of 78.9% in young and 84.9% in adults. These differences might be due to young animals were grazing around home as adult and older age groups graze/browse/ from grazing/browsing/areas far from the homesteads. This contributes young animals to a minimum rate of exposure by ticks since the distribution of ticks around homesteads are assumed to be less as compared to communal grazing grazing/browsing land (Rasmi et al., 2007).

Tick infestation was statistically (*p* < 0.05) associated with body condition of animals. The proportion of tick infestation was higher in poor body conditioned as compared to medium and good body conditioned animals. This is in agreement with the study conducted by Seid (2004) and Gedion et al. (2019). This difference might be poor body condition may cause reduced resistance which aggravates the susceptibility to tick infestation (Manan et al., 2007). Whereas reports of Yonas et al. (2020) and Jafer et al. (2017) recorded higher prevalence's in medium and good than poor body conditioned small ruminants.

In the present study, higher prevalence of Ixodid tick infestation in the mid land (62.88%), high land (43.18%) and low land (35.6%) agroecological systems were recorded. The present finding disagrees with the study conducted by Asnake et al. (2013) in three agroecological system of Southern Ethiopia who recorded tick infestation prevalence in small ruminant of as 2.61%, 0% and 97.95% in high land, mid land and low land, respectively. Similarly, this finding is in agreement with Abebe et al. (2011) in Tigray region where 57%, 61.6% and 37%, in low land, mid land and high land, respectively. The higher prevalence in mid land is recorded may be due to suitable climatic conditions (temperature, moisture, humidity and rainfall) that favour the survival and reproduction of ticks (Bersissa et al., 2012; Pegram et al., 1981).

In the present study, higher prevalence of Ixodid tick infestation was recorded in the wet season (61.11%) than in dry season (33.33%). This study is different from the study done by Mohamed et al. (2014) in and around Haramaya town, Ethiopia who recorded higher prevalence dry season (91.3%) than wet season (78%). This may be due to the difference in the distribution of rainfall where ticks need appropriate moisture and temperature for faster reproduction.

*Amblyomma variegatum* was the most abundant tick species with 18.2% prevalence in the present study. The result is slightly in agreement with 21.4% prevalence report by Kedir and Petros (2015) in Eastern part of Ethiopia; 24.5% prevalence by Jafer et al. (2017) in and around Dire Dawa and 15.96% prevalence by Abunna et al. (2012) in Bedelle District, Oromia Region. The prevalence of *A. varigatum* in this study was lower than 26% prevalence reported by Yishak et al. (2015) in Sodo zuria district. However, higher than the study conducted by Gedion et al. (2019) in and around Hawassa who recorded 6.25% prevalence of *A. varigatum* in small ruminants. The difference may be due to seasonal differences sampling where *A. variegatum* is not active throughout the year except during the favourable conditions during the wet season in highland areas of Ethiopia (Morel, 1980).

In the present study, *Rhipicephalus evertsi evertsi* was the second most abundant tick species (13.4%). The result of the present finding is in agreement with Alemu et al. (2014) reported 11.5% in Dembia Northwest Ethiopia and 11.2% by Hussein et al. (2018) in Hetosa district East Arsi. The prevalence of tick infestation in the current study was higher than previous works by Abunna et al. (2012), who reported 2.44% in Bedelle District, Oromia Region and Gedion et al. (2019) in and around Hawassa who recorded 1.03% in small ruminants. However, this species in the present study was lower than 38.5% prevalence reported by Yishak et al. (2015) in Sodo zuria district, 34.9% by Kedir and Petros (2015) in Selected Districts of Fafen. This tick species didn't show any apparent preference for particular altitude, rainfall or seasons to reproduce and survive. *Rhipicephalus evertsi evertsi* is active mainly during the summer but survived throughout the year in warmer climatic ones (Tiki & Addis, 2011).

In this study, the overall prevalence of tick‐borne haemopathogens in small ruminants was found to be 4.29% which was comparable with 6.3% prevalence reported by Setotaw et al. (2014) in Central Ethiopia and 3.03% prevalence reported by Ademola and Onyiche (2013) in Bodija Abattoir, Ibadan, Nigeria. This report is lower than the prevalence of 57.6% reported by Ukwueze and Kalu (2015). The present results were also disagreeing with 24.3% reported by Dabi and Wale (2017) in and around Sebata town, Oromia regional state, Ethiopia. Similarly, this finding also disagrees with the study conducted by Egbe‐Nwiyi et al. (2018) in Nigeria who was recorded higher prevalence (13.5%) of tick‐borne pathogens. The variation may be due to low prevalence of haemoparasite vectors.

In the present study, the prevalence of tick‐borne haemopathogens in old age was higher (7.4%) than adult (4.2%) and young (1.7%) small ruminants. This finding disagrees with the study conducted by Dabi and Wale (2017) in Oromia regional state, Ethiopia who reported prevalence of 40.38%, 20.76% and 5.55% in young, adult and old ages respectively. The prevalence of tick‐borne haemopathogens in this study is in agreement with reports of Amina et al. (2017) in Balami Sheep from Maiduguri, Northeastern Nigeria, who reported the prevalence of tick‐borne haemopathogens in adult (14%) and young (4.4%). Similarly, Setotaw et al. (2014) recorded a prevalence of 2.3% and 3.9% tick‐borne haemopathogens in young and adult ages respectively. The reason for the differences in the reported prevalence rates might be due to differences in climatic or geographical variation of study areas, period of sample collections, sample size and breed variation of small ruminants.

There was a statistically significant (*p* < 0.05) difference between prevalence of tick borne‐heamopathogens and season of occurrence. Higher prevalence of tick‐borne haemopathogens was recorded in wet season (6.57%) than dry season (2.02%). This finding is in line with the study conducted by Taddese et al. (2013) in central Ethiopia who reported 4.35% prevalence in dry season. This result disagrees with previous study by Yismashewa (2005) in Decha Woreda, Southern Ethiopia and Seyoum (2007) in Kobo and Girana valley in Amhara region who reported higher prevalence in those areas during dry season where there could be available moisture at dry period.

## 5 CONCLUSION

The present study revealed there were higher tick infestation and tick‐borne haemopathogens on small ruminants in the study area. Older age, poor body condition, midland and wet season were significant risk factors for the occurrence of higher tick infestation. Sheep is the most affected species by ticks. Occurrence of tick‐borne haemopathogens was higher in wet than the dry season. *Amblyomma variegatum* and *Rhipicephalus evertsi evertsi* were the most commonly encountered tick species. *Anaplasma ovis, B. ovis* and *A. marginale* were the predominant tick‐borne pathogens encountered in the study area respectively. Effective tick control and strategic prophylactic treatments should be implemented to minimise effect of ticks and haemoparasites in small ruminants. Due attention should be given to older, poor body conditioned small ruminants at midland agroecology and wet season. Further detailed study on productivity and economic loss associated with ectoparasites and haemoparasites should be conducted. Furthermore, awareness creation for small ruminant producers in the community on the impact of tick and tick‐borne disease should be carried out.

## AUTHOR CONTRIBUTIONS

**Habtamu Tamrat**: Formal analysis, methodology, supervision, validation, writing – original draft, writing – review and editing. **Wudu Tagel**: Conceptualisation, data curation, investigation. **Negus Belayneh**: Methodology, writing – original draft, writing – review and editing.

## FUNDING INFORMATION

No funding was obtained for this study.

## CONFLICT OF INTEREST

The other authors declare no conflict of interest

### ETHICS STATEMENT

The authors confirm that the ethical policies of the journal, as noted on the journal's author guidelines page, have been adhered to. No ethical approval was required, as this study required no animal experiments

### PEER REVIEW

The peer review history for this article is available at https://publons.com/publon/10.1002/vms3.1080.

## Data Availability

The data that support the findings of this study are available from the corresponding author upon reasonable request
